# Intervention with Exogenous Melatonin Has No Adverse Effects on Cardiometabolic Parameters of Overweight Night Workers without Cardiometabolic Abnormalities

**DOI:** 10.1055/s-0046-1829852

**Published:** 2026-11-06

**Authors:** Patrícia Teixeira de Santana, Adriana de Sousa Duarte, Ananda Laís Felix Garrido, Pollyanna Pellegrino, Luciana Fidalgo Ramos Nogueira, José Cipolla-Neto, Claudia Roberta de Castro Moreno, Elaine Cristina Marqueze

**Affiliations:** 1 Pharmacy Department, Hospital Municipal de Cubatão, Cubatão, SPBrazil; 2 Teaching and Research Laboratory, Sociedade Brasileira Caminho de Damasco, São Paulo, SPBrazil; 3 Department of Physiology, Intituto de Ciências Biomédicas, Universidade de São Paulo, São Paulo, SPBrazil; 4 Department of Health, Life Cycles, and Society, Faculdade de Saúde Pública, Universidade de São Paulo, São Paulo, SPBrazil; 5 Stress Research Institute, University of Stockholm, StockholmSweden; 6 Department of Public Health, Post-Graduate Program in Public Health, Escola de Saúde, Universidade Federal do Rio Grande do Norte, Natal, RNBrazil; 7 Independent Research, São Paulo, SPBrazil

**Keywords:** melatonin administration, circadian desynchronization, cardiometabolic parameters, overweight, night workers

## Abstract

**Background:**

Night workers are chronically exposed to circadian disruption and melatonin suppression, conditions associated with an increased risk of cardiometabolic disorders.

**Objective:**

To determine whether exogenous melatonin administration impacts cardiometabolic parameters (glucose, glycated hemoglobin [HbA1c], insulin, total cholesterol, high-density lipoprotein [HDL], low-density lipoprotein [LDL], very low-density lipoprotein [VLDL], triglycerides, heart rates, and mean systolic and diastolic blood pressure) in overweight night workers without cardiometabolic abnormalities.

**Materials and Methods:**

A randomized, double-blind, placebo-controlled, crossover trial was conducted. Participants (n = 27) took 3 mg of fast-release melatonin or placebo orally for 3 months, 1 hour before bedtime, only on days off when they had nocturnal sleep, on an intermittent basis (every other day). Generalized estimating equations (GEEs) were used to analyze the cardiometabolic parameters.

**Results:**

Systolic blood pressure showed a significant effect of time ( *p* = 0.01). Bonferroni-adjusted post-hoc comparisons showed higher values after placebo than at baseline (mean difference: 5.82 mmHg; 95%CI: 2.02–9.62; adjusted *p* = 0.01), whereas the difference between melatonin and placebo was not statistically significant (adjusted *p* = 1.00). No statistically significant effects of time were detected for the other cardiometabolic parameters.

**Conclusion:**

Melatonin was not associated with significant changes in cardiometabolic parameters compared with placebo in overweight night workers.

## Introduction

The demand for uninterrupted services in modern society has increased exposure to shift work, a modality associated with increased risk of developing chronic diseases, including cardiometabolic disorders. According to Moreno et al., 1 the main mechanisms explaining these increased risks include melatonin suppression and circadian desynchronization, both of which are commonly observed among night workers, although their extent may vary across individuals.

As reported by Imenshahidi et al., 2 the role of melatonin in preventing and improving cardiometabolic parameters has been previously demonstrated in several studies involving animal models. However, few studies have evaluated the impact of exogenous melatonin administration on cardiometabolic health in humans, and no investigations of this kind are available in overweight night workers without cardiometabolic abnormalities.

In a previous investigation by our group, 3 an approximate 20% reduction in circadian desynchronization was found after exogenous melatonin administration among the same night workers assessed in the present study. Furthermore, the use of melatonin by individuals who had high circadian desynchronization, as seen in early chronotypes, promoted a reduction in body weight, body mass index (BMI), waist circumference, and hip circumference, without any change in caloric intake or physical activity level of participants. 3 In other words, exogenous melatonin administration to night workers, who are chronically desynchronized and have melatonin suppression, proved beneficial for their health.

In this context, the present study hypothesized that exogenous melatonin can be safely administered to overweight night workers without cardiometabolic abnormalities. Therefore, a randomized, double-blind, placebo-controlled crossover trial was conducted to determine the impact of exogenous melatonin administration on the cardiometabolic parameters of overweight night workers.

## Materials and Methods

During the study, 238 nursing professionals were invited to participate. Of these, 192 were excluded: 152 did not meet the inclusion criteria and 40 declined to participate. Thus, 46 participants were randomized. Furthermore, 10 participants were lost during randomization, 8 due to incomplete questionnaire data and two due to incomplete biochemical data. During the first allocation phase, a further 9 participants were lost due to starting a second night-shift job, pregnancy, changes in work schedule, or leaving their employment. Thus, 27 participants remained for the second allocation phase.

All nurses worked a scheme of 12 ×36 hours (12 hours night shifts, from 7:00 pm to 7:00 am , followed by 36 hours off). The study participants took 3 mg of fast-release melatonin or placebo orally for 3 months, 1 hour before bedtime. The 3 mg dose was selected based on previously published clinical studies using doses ranging from 3 to 10 mg. Tablets were taken only on nights off between shifts, meaning, on those days participants slept the night at home. After this 3-month stage, participants switched groups.

Assessments were performed at baseline and again within the last 10 days of the first and second stages of the intervention. Blood samples were collected after 12 hours of fasting and after at least 6 hours of a night's sleep at home, to determine levels of plasma glucose, glycated hemoglobin, insulin, high- (HDL), low- (LDL), and very low-density (VLDL) lipoprotein, and total cholesterol, as well as triglycerides. The 6-hour period represented the minimum required sleep duration; participants who slept longer than 6 hours were assessed without adjustment for the additional sleep time. Systolic and diastolic blood pressure, along with heart rate, were measured in one arm after 15 minutes at rest during night-shift work. Three blood pressure readings were taken at 2-minute intervals, and the mean of all three measurements was used as the final value. Systolic and diastolic blood pressure were analyzed as continuous variables, and no categorical classification was applied.

This clinical trial complied with ethics requirements. All participants provided written informed consent to participate in the study. The trial was registered on the World Health Organization's International Clinical Trials Registry Platform and on the Brazilian Registry of Clinical Trials (Registro Brasileiro de Ensaios Clínicos, ReBEC, in Portuguese, under number RBR-6pncm9).. 3

## Results

Participants had a mean age of 37.1 years, with a standard error (SE) of 5.9 years. Furthermore, 63% were married, 51.9% nurses, and 48.1% nursing technicians. Mean time working night shifts during the lifespan was 9.2 (SE = 6.4) years, and mean BMI at baseline was 29.9 (SE = 3.3) kg/m ^2^ .

Systolic blood pressure differed significantly across time points. Bonferroni-adjusted post hoc comparisons showed it was higher after placebo than at baseline, with a mean difference of 5.82 mmHg and a 95%CI of 2.02 to 9.62 (adjusted *p* = 0.01). No statistically significant difference was observed between melatonin and placebo (mean difference: -1.55 mmHg; adjusted *p* = 1.00). No statistically significant changes over time were observed for the other cardiometabolic parameters ( Table 1 ).

**Table 1:** Effect of melatonin and placebo administration on the cardiometabolic parameters of overweight night workers (n = 27)

Variables	Baseline	Melatonin	Placebo	GEE *p* -value
	Mean (SE)	Mean (SE)	Mean (SE)	
Resting HR (bpm)	78.8 (2.18)	80.5 (2.18)	80.7 (2.21)	0.63
SBP (mmHg)	120 (2.70) ^a^	124 (2.70)	126 (2.70) ^a^	**0.01**
DBP (mmHg)	79.6 (2.46)	81.2 (2.46)	82.8 (2.46)	0.27
Glucose (mg/dL)	95.3 (2.18)	94.4 (2.18)	94.8 (2.18)	0.80
HbA1c (%)	5.30 (0.08)	5.41 (0.08)	5.37 (0.08)	0.25
Basal insulin (μUI/m)	14.6 (1.77)	13.1 (1.77)	16.1 (1.77)	0.35
Total cholesterol (mg/dL)	190 (8.88)	185 (8.86)	189 (8.88)	0.37
LDL (mg/dL)	110 (6.34)	107 (6.34)	111 (6.34)	0.70
HDL (mg/dL)	52.7 (2.58)	52.6 (2.59)	51.4 (2.57)	0.72
VLDL (mg/dL)	21.5 (1.53)	20.7 (1.53)	22.6 (1.53)	0.21
Triglycerides (mg/dL)	112 (11.7)	109 (11.7)	122 (11.7)	0.25

**Abbreviations:** DBP, diastolic blood pressure; GEE, generalized estimating equation; HbA1c, glycated hemoglobin; HDL, high-density lipoprotein; HR, heart rate; LDL, low-density lipoprotein; SBP, systolic blood pressure; SE, standard error; VLDL, very low-density lipoprotein. **Notes:** Values presented were calculated using the GEE. ^a^ Bonferroni-adjusted post-hoc comparison between baseline and placebo: mean difference = 5.82 mmHg (95%CI: 2.02–9.62; adjusted *p* = 0.01).

## Discussion

The present study investigated the effects of intermittent exogenous melatonin administration over a 3-month period on cardiometabolic parameters in overweight night workers without baseline cardiometabolic abnormalities. Systolic blood pressure was significantly higher after placebo administration than at baseline. No statistically significant difference in systolic blood pressure was observed between melatonin and placebo. No statistically significant changes were observed over time in the other cardiometabolic parameters assessed.

According to the literature, exogenous melatonin can be beneficial for cardiometabolic parameters. Bazyar et al. 4 investigated its effect on the risks of cardiovascular diseases and on anthropometric indices in patients with type 2 diabetes mellitus. The participants used 3 mg of melatonin 1 hour before bedtime for 8 weeks. Exogenous melatonin effectively controlled blood pressure levels and improved anthropometric indices in diabetic patients. Moreover, in a systematic review and meta-analysis of randomized clinical trials, Akbari et al. 5 evaluated its effect on blood pressure in patients with metabolic disorders. The studies reviewed used melatonin doses of 2 to 10 mg (average 5 mg) for a period of 4 to 12 weeks (average 8 weeks). Melatonin supplementation was found to significantly reduce systolic and diastolic blood pressure in patients with metabolic disorders.

In a meta-analysis 6 to determine the effects of exogenous melatonin at doses above 3 mg on glycemic control, for an intervention period exceeding 4 weeks, the authors found a significant decrease in fasting blood glucose and an increase in the quantitative insulin sensitivity check index (QUICKI). However, no effect on glycated hemoglobin and basal insulin was evident. 6 Similarly, a double-blind, randomized clinical trial 7 involving 36 diabetic patients (11 men and 25 women, aged 46–77 years), in which 2 mg of prolonged-release melatonin was administered for 3 weeks, 2 hours before bedtime, found no effect on glycemic parameters, including glucose, insulin, and glycated hemoglobin. Nevertheless, a significant decline in glycated hemoglobin levels was observed when the study period was extended to 5 months in 29 patients (10 men and 19 women). The authors concluded that prolonged-release melatonin significantly lowers glycated hemoglobin levels over the long term at low doses. 7

A meta-analysis conducted by Mohammadi-Sartang et al., 8 investigating the effect of exogenous melatonin on plasma lipid levels, found a decrease in triglyceride and total cholesterol levels after administration of 8 mg melatonin in studies with ranges over 4 to 56 weeks. However, no significant effects on HDL or VLDL-cholesterol levels were detected. The authors underscored that the effect of melatonin on triglycerides is clear at higher doses, irrespective of the length of time administered. In a more recent meta-analysis, triglyceride levels were found to decrease after melatonin administration over a short period (8 weeks) at doses exceeding 8 mg. A decrease in LDL-cholesterol level was also found when melatonin was taken for over 8 weeks. Conversely, no improvement in triglyceride level was evident after melatonin administration over an 8-week period at doses below 8 mg. 9 Although these two reviews concluded melatonin dosage should exceed 8 mg to be effective for improving lipid levels, there is conflicting evidence with respect to length of administration, with no current consensus. This lack of clarity may be due to differences in the samples studied for individual and environmental characteristics.

In a recent study involving four patients with melatonin deficiency secondary to surgical removal of the pineal gland, endogenous melatonin was replaced by administering 3 mg of melatonin. After administration, all patients exhibited lower total cholesterol and triglyceride levels and increased brown adipose tissue activity, but without any significant effects on body weight. 10

Previous studies have suggested that exogenous melatonin may positively influence cardiometabolic parameters in individuals with certain pathologies and/or chronic diseases. In the present study, participants were overweight night workers without baseline cardiometabolic abnormalities. Systolic blood pressure increased significantly after placebo administration compared with baseline, whereas no statistically significant difference was observed between melatonin and placebo. No statistically significant changes over time were observed in the other cardiometabolic parameters assessed. These findings are consistent with the study's hypothesis that intermittent melatonin administration does not adversely affect cardiometabolic parameters in overweight night workers without related abnormalities. Further studies may contribute to expanding the evidence on the cardiometabolic effects of melatonin in night workers.
